# Semi-supervised method for biomedical event extraction

**DOI:** 10.1186/1477-5956-11-S1-S17

**Published:** 2013-11-07

**Authors:** Jian Wang, Qian Xu, Hongfei Lin, Zhihao Yang, Yanpeng Li

**Affiliations:** 1 School of Computer Science and Technology, Dalian University of Technology, Dalian, China

## Abstract

**Background:**

Biomedical extraction based on supervised machine learning still faces the problem that a limited labeled dataset does not saturate the learning method. Many supervised learning algorithms for bio-event extraction have been affected by the data sparseness.

**Methods:**

In this study, a semi-supervised method for combining labeled data with large scale of unlabeled data is presented to improve the performance of biomedical event extraction. We propose a set of rich feature vector, including a variety of syntactic features and semantic features, such as N-gram features, walk subsequence features, predicate argument structure (PAS) features, especially some new features derived from a strategy named Event Feature Coupling Generalization (EFCG). The EFCG algorithm can create useful event recognition features by making use of the correlation between two sorts of original features explored from the labeled data, while the correlation is computed with the help of massive amounts of unlabeled data. This introduced EFCG approach aims to solve the data sparse problem caused by limited tagging corpus, and enables the new features to cover much more event related information with better generalization properties.

**Results:**

The effectiveness of our event extraction system is evaluated on the datasets from the BioNLP Shared Task 2011 and PubMed. Experimental results demonstrate the state-of-the-art performance in the fine-grained biomedical information extraction task.

**Conclusions:**

Limited labeled data could be combined with unlabeled data to tackle the data sparseness problem by means of our EFCG approach, and the classified capability of the model was enhanced through establishing a rich feature set by both labeled and unlabeled datasets. So this semi-supervised learning approach could go far towards improving the performance of the event extraction system. To the best of our knowledge, it was the first attempt at combining labeled and unlabeled data for tasks related biomedical event extraction.

## Background

Recently, some models of biomedical event extraction have aroused substantial interest in bioinformatic domain. The expressive event representation captures extracted knowledge as structured, recursively nested, typed associations of arbitrarily many participants in specific roles [1]. In other words, event extraction refers to tasks the purpose of which is extracting information beyond the entity level. Commonly, a task of event extraction contains identifying some actions and relations between entities [2]. There is a trigger which is one or more tokens in every event. Different types of events can share the same triggers and arguments [3]. The complexity of the task can be demonstrated in the example as: "RFLAT-1: a new zinc finger transcription factor that activates RANTES gene expression in T lymphocytes". Table 1 shows that the participating system, given the two proteins "RFLAT-1" and "RANTES", needs to generate three appropriately nested events.

**Table 1:** Example annotation

ID	E1	E2
Type	Gene_expression	Positive_regulation
Trigger	Expression	Activates
Theme	RANTES	E1
Cause		RFLAT-1

At present, the proposed approaches to extract events can be divided into 2 main groups: namely rule-based and machine learning (ML)-based extraction methods. Rule-based event extraction systems consist of a set of rules that is manually defined or generated from training data. ConcordU System is an event extraction system that is the best rule-based system in BioNLP '09 [4]. ML-based systems model event extraction tasks as a classification problem. In these systems, pre-selected candidate event triggers are classified as true event triggers or not. Riedel et al. used the Markov Logic approach, a statistical relational learning language, and defined the global model declaratively [5]. UTurku system is generic and capable of producing predictions for every BioNLP Shared Task, during which process, ML is used intensely, especially SVM (Support Vector Machine), for entity recognition, entity typing and event extraction [6]. Simultaneously, this system can separate event extraction into multiple classification tasks, individually detecting the trigger words defining events, and the arguments that describe which proteins or genes take part in these events [7,8]. In addition, the FAUST system which is a variant of the model, exploits several stacking models for combination using as base models the UMass dual decomposition and Stanford event parsing approaches [9]. Its advantage stems from the fact that it uses predictions of the Stanford system and hence performs model combination [10]. David et al. [11] attempted to combine the word sense disambiguation (WSD) with a CRF approach for event trigger recognition. As a result, they gain a high recall in the detection of trigger words.

These aforementioned systems are all based on labeled data and their supervised learning algorithms have been developed and extensively studied. However, these methods are affected by data sparseness, especially when the size of training corpus is too small to find enough information to assign proper weights to those low-frequency or out-of-vocabulary (OOV) features [12]. For example, multi-word expressions are seldom included in the training feature set for their high sparseness, and they probably should be ignored by experiment designers, but in fact, these filtered features may have strong classification ability in the global datasets. Fortunately, there is a large pool of unlabeled data containing much of potential information in the biomedical event extraction domain, such as data in PubMed. We recently proposed a biomedical event extraction method, which generates new features by estimating proper weights for low-frequency features from the labeled and unlabeled data and solves the problem of data sparseness [13]. This presented paper based on a semi-supervised learning strategy is an expansion upon [13]. Compared with [13], the expansions of this study are as follows:

1) We made a more detailed analysis of the unlabeled data, finding the fact that there is much noise in it. Then we filtered the unlabeled data properly and obtained a more specific corpus for biomedical event extraction.

2) We utilized the deep parser Enju [14] to output the predicate-argument structures, which contain lots of useful information ignored in our previous study.

3) The framework of the new feature vector was established by increasing the deep parsing features explored by Enju.

As a result, the overall performance of our biomedical event extraction method is improved: an improved F-score from 66.3% to 67.6% is achieved in the stage of the trigger detection. Meanwhile, the F-score (54.17%) of our event extraction system have outperformed that (53.30%) of the Uturku system, the state-of-the-art system in biomedical event extraction.

## Methods

**Figure 1:**
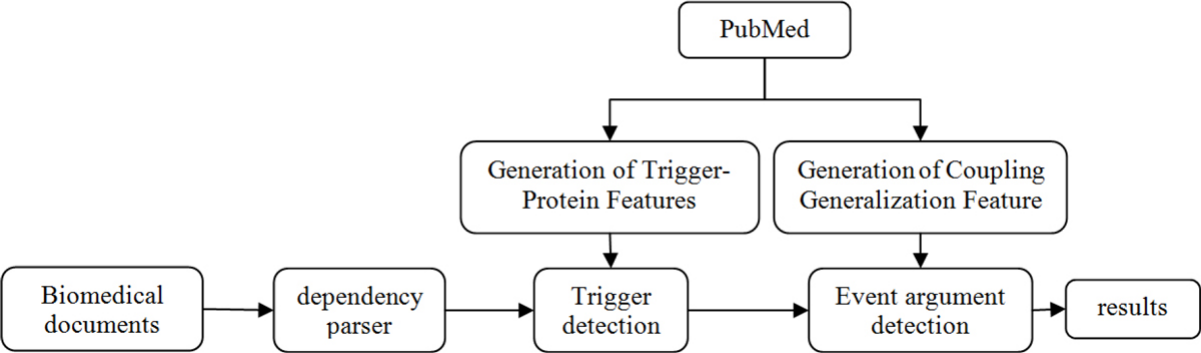
**Processing pipeline for extracting events from biomedical documents**.

Events can have an arbitrary number of participants with specified roles, making it possible to capture associations and statements where some participants occur in varying roles or are only occasionally mentioned. Our system extracts a 4-tuple representation of events that includes a type, trigger, theme and cause (Table 1). This representation was chosen to closely match the way important events are typically mentioned in biomedical events. An overview of the various components of our system for extracting events is presented in Figure 1. Given a raw stream of documents, our system extracts named triggers in association with arguments and nested event which are involved in the event. The study follows the process of the UTurku system for the extraction of events, which contains trigger recognition, argument detection and post-processing steps [6,15].

We use the shallow and deep syntactic analysis tools to analyze every sentence in the corpora, and on this basis we make the three major processing steps: The named triggers are recognized; the extracted events are categorized into types; the arguments belonging to the event are extracted.

### Pre-processing

**Figure 2:**
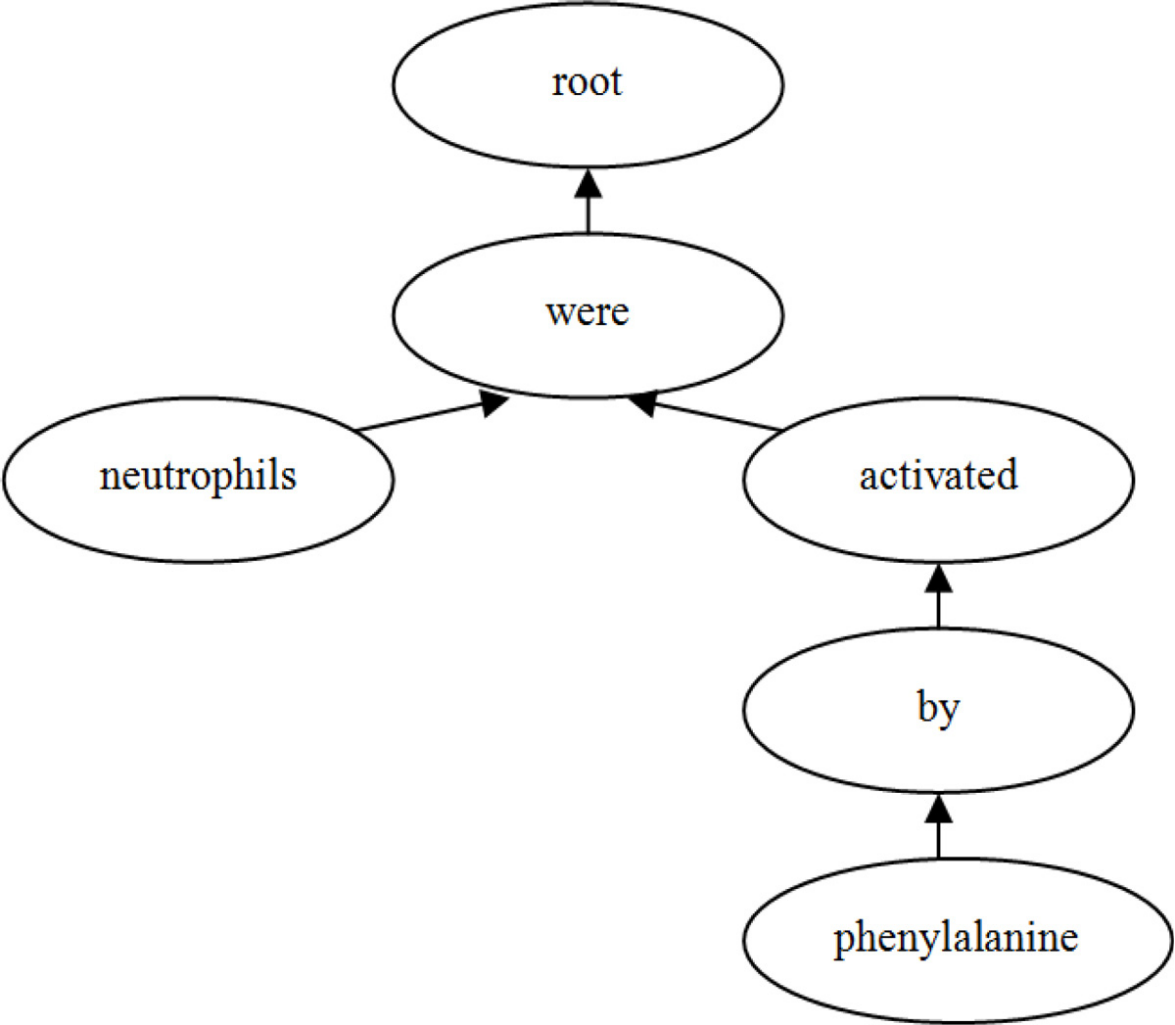
**A dependency tree for the sentence "neutrophils were activated by phenylalanine"**.

Firstly, we pre-process the corpora by making a full dependency analysis with the GDep dependency parser [16][17] and the deep parsing tool Enju[14]. The parsers can output rich syntactic and semantic structure information which can be treated as features for machine learning. A dependency structure is a representation that denotes grammatical relations between words in a sentence (Figure 2). A set of rules maps a parse tree to a dependency structure. For example, subjects are dependent on their verbs and adjectives are dependent on the nouns they modify.

### Trigger detection

Trigger word detection is put as a token labeling problem. It is different from argument detection: we treat trigger detection as named trigger recognition, and assigne an event class or a negative class to each token at a time, so trigger detection can be seen as a multi-class task which is resolved by support vector machine (SVM) classifier. The classifier is trained from the labeled and unlabeled data. We apply a wide array of syntactic and semantic features in our experiment. Among the large feature set, we call the types of features derived from the parsers as basic features, such as token features, N-gram features, walk subsequence features, predicate argument structure (PAS) features. Besides, we create some new features like triggerprotein features (TPF) from unlabeled data. There is a corresponding relationship between trigger and protein. Hence, in trigger recognition, we simply need to find the trigger in the sentence that contains the assigned protein. Furthermore, a trigger can be seen as "belonging" to an assigned protein. Given this kind of relation information, a trigger can be detected by looking up a corresponding protein in the sentence but without the real corresponding pairs. Here we try to use the statistics extracted from unlabeled data to estimate the distribution of these pairs.

**Figure 3:**
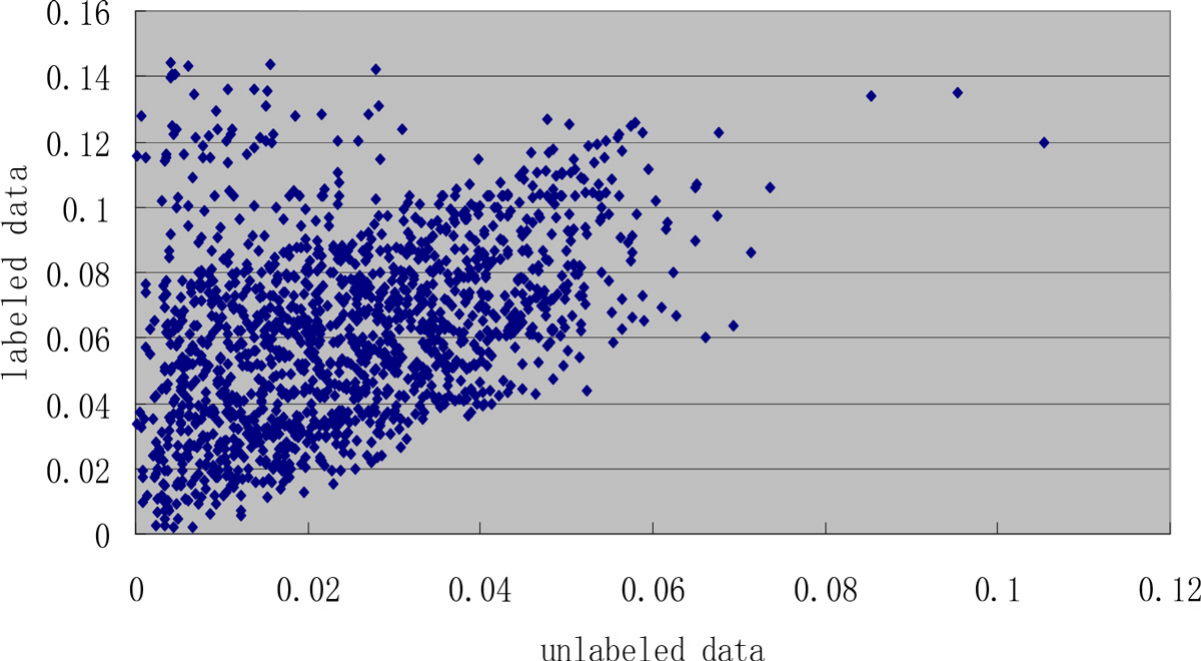
**The scores of the entity pairs in labeled and unlabeled data**.

The training set of GE task in BioNLP Shared Task 2011 (BioNLP 2011, hereafter) is considered as labeled data, while the PubMed abstracts (up to 2009) are unlabeled data. The entity pairs are obtained from labeled data while the statistics are extracted from both labeled and unlabeled data. First of all, the scores of pairs in labeled and unlabeled data should be separately computed by a score equation which will be introduced in the section of "Event Feature Coupling Generalization Method". The scores' distribution tendency is shown in Figure 3, from which we can see clearly that most pairs have similar scores in labeled and unlabeled data in spite of a small quantity of pairs not being in unlabeled data. To obtain the high precision, we could set a proper threshold to filtrate the low scores. But it might lose some "special" pairs whose distribution in labeled data is totally different from that in unlabeled data. Therefore, instead of setting a threshold that can be seen as a "hard" strategy, we present a "soft" one, namely, adding the scores into feature space in order that the high scores still have a strong impact, whereas other features can help models to make up this bias.

To recognize the event triggers and arguments, we choose the following 4 types of features as the basic features (BFs) in our feature vector set:

1) Token features: Token features include the presence of words shown around the tokens, the presence of tokens and POS tags, and the presence of stems analysed by GDep parser.

2) N-gram features: In trigger recognition, N-gram features include the N-grams (n = 1, 2, 3, 4) of words in 4-word-window around candidate word. In edge detection, N-gram features include the N-gram (n = 2, 3) of words (base form+ POS) and N-gram (n = 1, 2, 3, 4) of words between 2 entities.

3) Walk subsequence features: Walk subsequence features focus on different structure properties of vertex walk (v-walk) and edge walk (e-walk). A labeled relationship from a head to its modifier is a v-walk. Thus, a direct dependency relationship between two nodes is related. On the other hand, an e-walk shows the immediate dependency structure around a node. A word vertex walk (wv-walk) means that the node in vertex walks is a word, while a POS vertex walk (POSv-walk) means that the node is POS.

4) Predicate-argument structure features: A deep parser, like Enju, can usually produce sematic structure, such as a predicate-argument structure (PAS). This kind of structures is typically corresponding to events of interest and their arguments. Since PAS represents syntactic relations among tokens, we take the key word that is connected with more than 3 tokens as a PAS feature.

As shown in Table 2, we obtain a result by basic features and get another result by adding TPF. In particular, we improved optimal F-score by up to 67.6%. WSD system is a word sense disambiguation system for event trigger word detection [11]. Compared to WSD system, we obtain an 11% improvement in F-score by using the feature set including basic features and TPF.

**Table 2:** Results of trigger detection

	R(%)	P(%)	F(%)
Basic features	57.3	54.6	55.9
WSD system	70.2	52.6	60.1
Basic features +TPF	66.1	69.8	67.6

### Event argument detection

The present study addresses event argument detection with the task of predicting, for each trigger-protein or trigger-trigger pair, whether it corresponds to an actual instantiation of an event argument. Like the trigger detection, event argument detection is based on a multi-class SVM classifier. We generate examples, which are always directed from a trigger to another trigger or from a trigger to a named protein. We assign every example to 3 types of labels: theme, cause, or a negative denoting the absence of the relation between the two nodes.

To construct the sufficient feature set for event argument detection, we add a subset of new features to the feature set based on the basic features mentioned in trigger detection. We name the new type features as Coupling Generalization Features (CGFs), which are produced by a strategy named Event Feature Coupling Generalization (EFCG).

### Event Feature Coupling Generalization Method

Event Feature Coupling Generalization (EFCG) is a framework that can produce new features (CGFs) base on two types of original features: example-distinguishing features (EDFs) and class-distinguishing features (CDFs) [18,19]. EDFs are good at indicating the specific examples, while CDFs have the strong ability to distinguish the different classes. Here, we view the new CGFs as important features in the argument detection, whereas EDFs and CDFs are treated as prior features since they need to be generalized instead of being final representations of instances. In unlabeled data (u), the degree of relatedness between EDFs and CDFs is defined as the feature coupling degree (FCD), denoted by FCDt(u, e, c), which can be seen as a higher-level feature and that can enhance the performance of classification. The aim of EFCG method is the transformation of FCD into CGF, and the process of EFCG algorithm can be described as follows:

1) INPUT: the labeled data set D = {d_1_, d_2_, ..., d_m_}, the feature vocabulary F = {f_1_, f_2_, ..., f_n _}, the vector of labeled data set X = {x_1_, x_2_, ..., x_m _} ⊆R^n^, unlabeled data set U.

2) Select EDFs from F and the EDF set is denoted by E.

3) The EDFs in E will be mapped onto the higher-level concept set R by the map equation root (e).

4) Select CDFs from F as CDFs set, which are denoted by C.

5) Define the type T in FCD set to estimate the relatedness degree between EDFs and CDFs.

6) Based on unlabeled data U and FCD type T, the FCD value between every EDF and CDF is computed.

7) Create a new feature set G: G=R*C*T, and every CGF is corresponding to a tuple (r, c, t), where r ∈ R, c∈ C, t∈T.

8) For every instance d ∈ D, × ∈ × is converted to CGF vector v, in which every v_i _∈ v is computed by the following equation:

(1) $$v_{i}=v_{\left(r,c,t\right)}=\underset{root\left(e\right)=r}{\sum }band\left(e,d\right)*FCDt\left(U,e,c\right)$$

where i is index of CGF, which corressponds to the every tuple (r, c, t) in G. The function band (e,d) is 1 if the feature e apprears in the feature vector of instance d. 9) OUTPUT: CGF set G, where every CGF vector v = { v_1_, ..., v_m _} Rdim(R)*dim(C)*dim(T).

**Figure 4:**
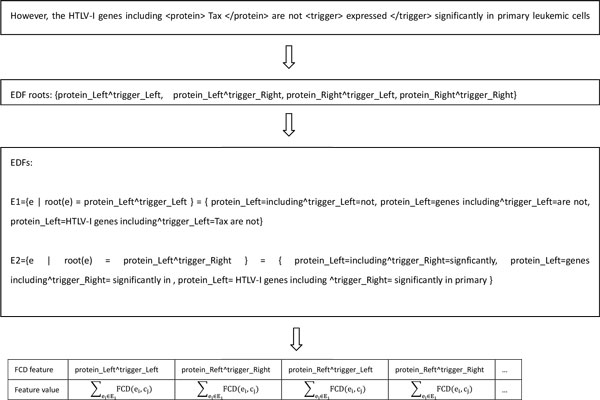
**An example of showing how EFCG generates new features**.

An example shows how FCG generates new features in Figure 4. Here, the transformed feature set instead of f generates EDFs and CDFs. For simplicity, in above algorithm, we suppose that all the possible unions of features are included in the F so that all EDFs and CDFs are restricted to be chose from this set. Often, however, only the subset of F can be used in supervised learning. Some features that have shown poor classification performance are ignored or assigned a little weight by algorithm designer. In EFCG framework, we also choose a subset of F as CDFs and EDFs set with extremely different evaluation criterion. Here, "good" features mean the strong classification performance of CGF that is generated by these features instead of the features themselves. In other words, "bad" features in unlabeled data may become "good" EDFs or CDFs, so that EFCG can make full use of the features unnoticed by supervised learning.

Here, CGF aims to find the relations between two target tokens (i.e. trigger-protein and trigger-trigger). In an example, given that words are shown by the under sequence: (0, ..., t_1_, ..., t_2_, ..., END), where t_1 _indicates the target token "target1", t_2 _indicates target token "target2", and END indicates the end of the sentence.

1) Words dictionary: LWD = {tokens of labeled data}, and UWD = {tokens of unlabeled data}.

2) word-level N-grams dictionary: LND = {2-3 grams of labeled data}, and UND = {2-3 grams of unlabeled data}.

3) General Region: GR = {Left_region, Inner_region, Right_region } = {[0, t_1 _-1], [t_1 _+1, t_2_-1], [t_2_+1, LAST]}.

4) Surrounding Region: SR = {T1_Left, T1_Right, T2_Left, T2_Right} = {[ t_1 _-4, t_1 _-1], [t_1 _+1, t_1 _+4], [t_2_-4,t_2_-1], [t_2_+1, t_2_+4]} - string surrounding "target1" or "target2" within a 4-token window.

5) Specific Location: SL = {m_From_T1, n_From_T2 | m = y - t_1_, n = y - t_2_, × ∈ [t_1 _- 5, t1 +5] ∪ [t2-5, t2+5]} - tokens or N-grams which show in specific location in SL with the 5-word window, and y is the index of the present string. Note that SL is slightly unlike from other features.

6) Conjunct Location: CL = {T1_orientation ^ T2_orientation ^ space | orientation ∈ {Left, Right}, space = orientation(t_2_- t_1_) ∈ {0,1, 2, 3, 4, (5~6), (7~9), (10~14), (15~19), (20~29), (30~39), (40~) } - union of local elements (2 locations near each targets) in SL and the token calculation between the 2 targets.

7) GL-BOW: Bag-of-words features extracted from LWD ×GL, for example, "Token_In_Left_Location=gene". These features neglect token location in the present string.

8) GL-bag-of-N-grams: features from LND × GL. It merely fertilizes the bag-of-tokens statement by bigrams and trigrams.

9) SL-N-grams environmental targets: features from LND × SL, for example, "Tar_Right=due to". N-grams are emphasized in the "indicating location".

10) SL-N-grams with particular offsets from 2 targets: features from LND × SL, which provides the knowledge of particular distances from the N-grams in SL. For example, "...pro due to tri...", some of SL-N-grams can be "1_From_Tar= deal", "2_From_Tar=to","1_From_Tar=to" and "2_From_Tar=due to". This feature provides more particular information than SL.

11) Conjunct Location N-grams: features from LND × CL. The feature set is the union of a subset of features in SL and the space of 2 targets. It can provide the lexical knowledge around both targets. However, it is more particular than SL. Thus, data sparseness is more likely in these features.

We select features as CDF by the following equation:

(2) $$score\left(feature\right)=\frac{{\left(t*k-m*n\right)}^{2}}{\left(t+m\right)\left(k+n\right)}$$

where *t, k, m, n *are the counts of 4 kinds of instances in the labeled data as shown in Table 3.

**Table 3:** Representative of variable

	Positive	Negative
Containing feature	t	m
Containing no feature	n	k

We select some features with high scores as CDF, the number selection of which will

be discussed in the section of results. Then we consider one type of measure:

(3) $$score\left(e,c\right)=\frac{{\text{log}}_{10}\left(co\left(e,c\right)+b\right)}{{\text{log}}_{10}(count\left(e\right)+b*{\text{log}}_{10}\left(count\left(c\right)+b\right)}$$

where *e *is an EDF, *c *is a CDF, and *co(e, c) *is the co-occurrence count of *e *and *c*. After a cross validation, we assign 1 to the smoothing factor *b*. We log the computation to avoid high score in a huge large data. This measure can be treated as a modification of Pointwise Mutual Information (PMI for short).

### BioNLP 2011 task dataset

Similarly to LLL(Learning Language in Logic) and BioCreative, the BioNLP 2011 also addresses bio-IE, but uses a decisive step further toward finer-grained IE. The BioNLP 2011 task focuses on the detailed movement of molecules, specialty on biomolecular events. It includes 4 main tracks representing fine-grained bio-IE. Genia task, as one of them, preserves the task definition of BioNLP 2009, which was arranged based on the Genia corpus. Therefore, the provided data are composed of two collections: the abstract collection, identical to the BioNLP 2009 data, and the new full paper collection. In the following description, BioNLP 2011 mentioned all means Genia task in BioNLP 2011.

The BioNLP 2011 task data were generated from the GENIA event corpus, in which training data is derived from the publicly available event corpus. Event types mentioned in the task are show in Table 4. Considering given to their value and the count of labeled instance in the GENIA corpus, 9 event types are chosen from the GENIA ontology. For each event type, the primary and secondary arguments to be extracted with an event are defined. For example, a phosphorylation event is primarily extracted with the protein to be phosphorylated. As secondary information, the specific site to be phosphorylated may be extracted. From the perspective of calculation, the 9 types stand for several levels of complexity. From a view of primary arguments, the first 5 event types in Table 4 are classified as simple event types, requiring only one argument. The binding and regulation types are more complex: Binding requires detection of more than one number of arguments, while regulation requires detection of recursive event structure.

**Table 4:** List of trigger types

Event Type	Primary Argument
Gene expression	Theme(Protein)
Transcription	Theme(Protein)
Protein catabolism	Theme(Protein)
Phosphorylation	Theme(Protein)
Localization	Theme(Protein)

Binding	Theme(Protein)+

Regulation	Theme(Protein/Event), Cause(Protein/Event)
Positive regulation	Theme(Protein/Event), Cause(Protein/Event)
Negative regulation	Theme(Protein/Event), Cause(Protein/Event)

### LibSVM tool

**Figure 5:**
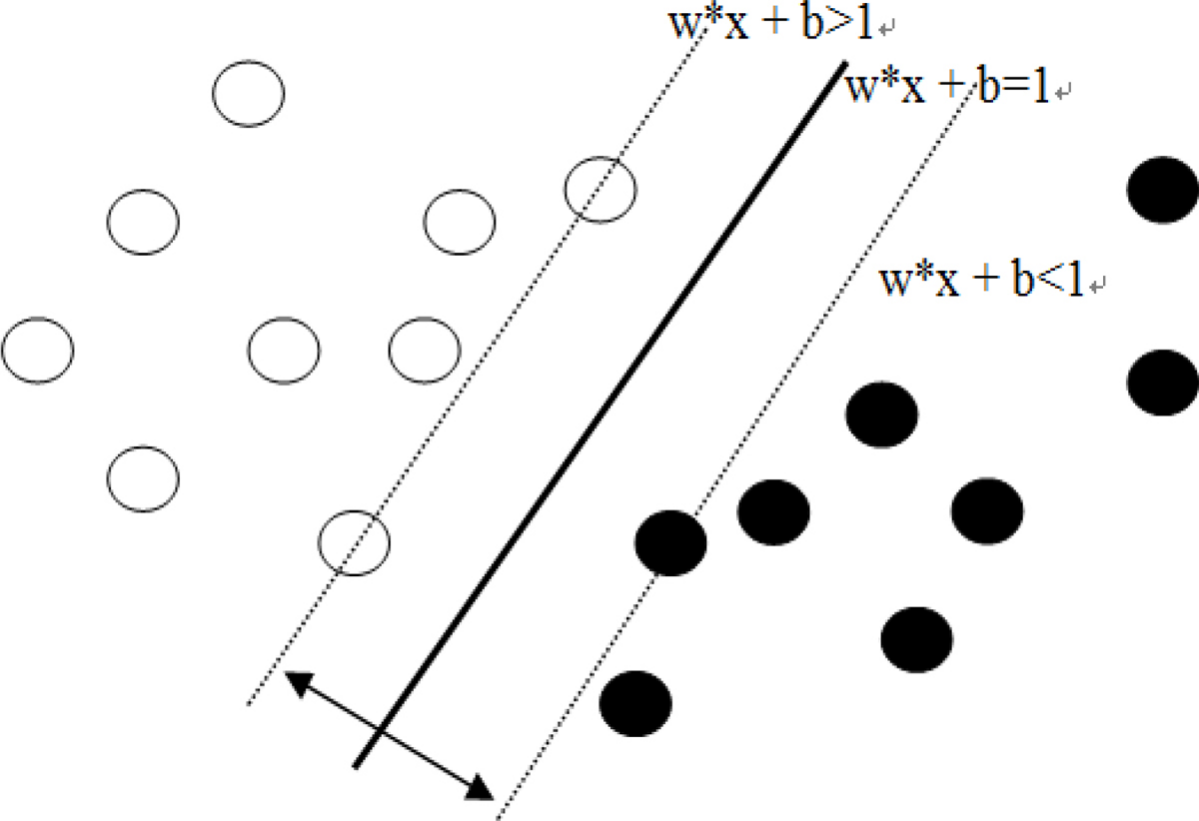
**Overview of Support Vector Machine**.

An SVM multi-class classifier, which proved to have ability of good classification, is used for the experiment. The LibSVM tool [20] which is one of the best multi-class SVM tools presently available is used. The goal of SVMs is to find a hyperplane that separates the positive examples from the negative examples. With SVMs, rather than defining some underlying event space, we must instead define a set of feature functions that take examples as input and produce what is known as a feature value. Notice that different feature functions will result in different embeddings. Since SVMs find a hyperplane that separates the data according to classes, it is important to choose feature functions that will help discriminate between the different classes. In order to avoid overfitting, SVMs choose the hyperplane that maximizes the separation between the positive and negative data points. This selection criterion makes sense intuitively, and is backed up by strong theoretical results as well. As shown in Figure 5, the optimal hyperplane with maximum margin can be obtained by solving the following quadratic programming.

(4) $$\text{min}\frac{1}{2}∥w∥+\;C\overset{len}{\underset{j}{\sum }}\xi j$$

$$\begin{array}{c} s.t.\;y_{j}\left(w\cdot x_{j}+b\right)\geq 1-\xi_{j}\\ \xi_{j}\geq 0 \end{array}$$

Here, C is the constant while ζ_j _is called a slack variable. Then, the optimal hyperplane is showed as follows:

(5) $$f\left(x\right)=sign\left(\overset{len}{\underset{j}{\sum }}\alpha_{j}y_{j}K\left(x_{j},x\right)+b\right)$$

where α_j _is the lagrange multiplier corresponding, and K(x_j _,x) is called a kernel function.

Multi-class SVM works by training K (K>2) classifiers. When training the Kth classifier, the Kth class is treated as the positive class and all of other classes are treated as the negative class. That is, each classifier treats the instances of a single class as the positive class, and the remaining instances are the negative class. We use the linear kernel and set the parameters of shrinking and probability estimates both to 1 in our method, while other parameters are set to default.

## Results

### CDF number selection

**Figure 6:**
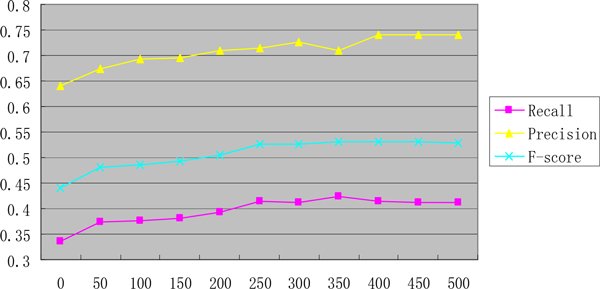
**The performance of different number of EDF**.

In the experiment, we first scrutinize and adjust parameters in our method. To observe how CDFs affect the result, we set the number of CDFs from 0 to 500 by increment of 50 every time. The precision, recall and F-score influenced by different number of CDFs are shown in Figure 6. With the increase in the number of CDFs, the F-score rises in general, indicating that a high number of CDFs contain rich potential classification information. However, above 250, the F-score changes little. Here, we choose 400 CDFs for our experiment.

### Effectiveness of unlabeled data

**Figure 7:**
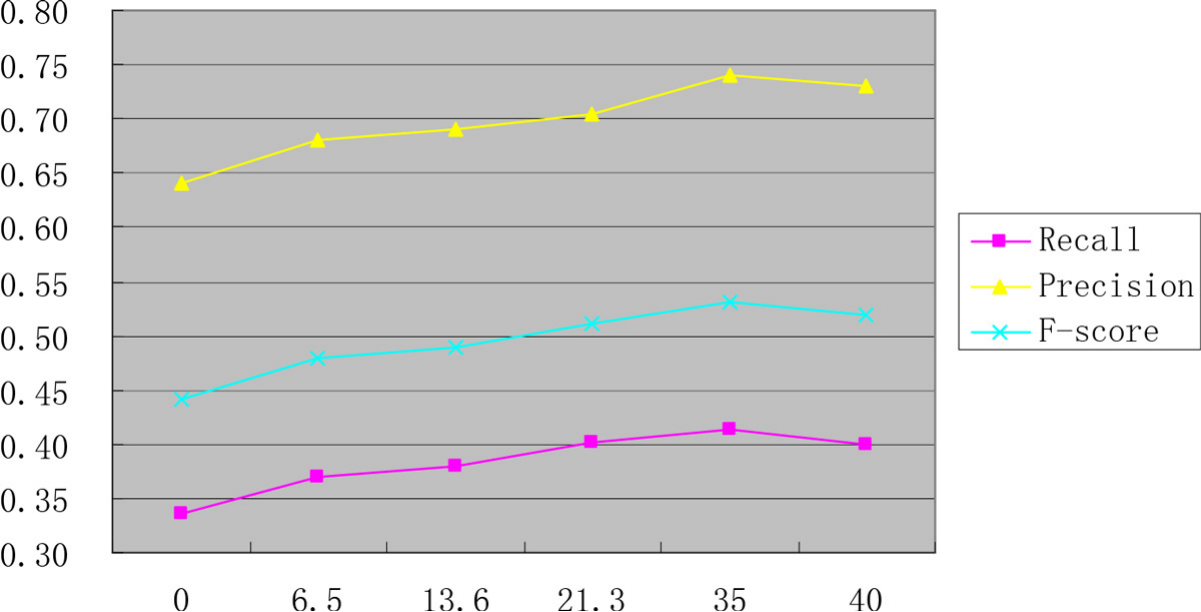
**The performance of different size of unlabeled data**.

Another experiment is presented to find the optimal size of unlabeled data. As shown in Figure 7, the larger the size of unlabeled data, the higher the F-scores. However, we argue that it more significant to find which kind of features from unlabeled data can contribute more useful information.

**Figure 8:**
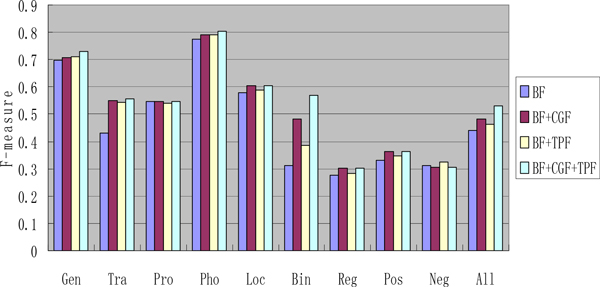
**The F-score with different features**.

The features extracted from labeled data are considered as basic features (BF). As shown in Figure 8, CGF and TPF are added to the experiment one by one. Stated broadly, the results show that both CGF and TPF can help to improve the performance significantly. However, in complex events (regulation, positive regulation and negative regulation event), either CGF or TPF is useless. Specifically, adding CGF makes performance worse in negative regulation events. This mainly because regulation (including positive and negative regulation) is a directed relation and the roles of the participants are different. Therefore, their information is complex and it is difficult to explore these features accurately by way of unlabeled data. Finding more effective features for the regulation event is our future work. According to Figure 8, we find out that the performance in binding event improves significantly. We argue that the rich information of binding event that labeled data lacks is explored from unlabeled data by our method.

### Performance compared with other approaches

As shown in Table 5, there are evaluation results of different system in dataset. Some notable figures are emphasized in bold. We reach the 3^th ^place in BioNLP 2011, which indicates that our system is still among state-of-the-art systems for bio-event extraction.

**Table 5:** Performance of different systems

	R	P	F
FAUST	49.41	64.75	**56.04 **
Our system	51.97	56.56	**54.17 **
UTurku	49.56	57.65	53.30

As we mention above, we follow the pipeline of the Uturku system and explore the new features from unlabeled data by our semi- supervised method. The F-score of our system is higher than that of the Uturku system. That is, based on the Uturku system, our system explores more information from unlabeled data to solve the sparseness problem. The improvement of performance indicates that combining labeled and unlabeled data is useful for biomedical event extraction.

From a comparative perspective, the F-score of our system is lower than that of the FAUST system, which obtained the best performance in BioNLP 2011. Since the two systems utilize different method, the semi-supervised method still has lots of room for improvement.

## Conclusions

Supervised methods for biomedical event extraction are often affected by data sparseness. In this paper, we firstly combined labeled with unlabeled ones to solve the problem effectively. From unlabeled data, we could explore the information that labeled data lacks. Through the Event Feature Coupling Generalization (EFCG) strategy, the classified capability of the model was enhanced to improve the performance of biomedical event extraction.

The major contributions of this study can be written as follows:

1) By our method, many opportunities for generating new features is created for this task, since the results indicate that a lot of sparse features filtered by supervised learning can perform well in our system.

2) We are the first to use semi- supervised method to resolve the task of biomedical event extraction. We explored several useful types of features and get state-of-the-art performance in BioNLP 2011 datasets.

3) By taking analyze for the different features in semi-supervised learning we find some interesting results.

4) Our results indicate that our method based on semi-supervised learning can performance well on biomedical event extraction.

For future work, it is extremely important to identify which kinds of unlabeled data

are more suitable to biomedical event extraction and contribute more to the performance. Finally, the tradeoff between recall and precision is also a topic for future research.

## Competing interests

The authors declare that they have no competing interests.

## Authors' contributions

JW carried out the identification of biomedical event extraction studies, participated in the design of the experiments and draft part of the manuscript. QX carried out the unlabeled data studies, proposed the method of combining labeled and unlabeled data, and draft the manuscript. HFL guided the study of the related work and conceived the experiments. ZHY and YPL participated in the implementation and evaluation of the system. All authors read and approved the final manuscript.

## Declarations

The publication costs for this article were funded by the corresponding author.

This article has been published as part of *Proteome Science *Volume 11 Supplement 1, 2013: Selected articles from the IEEE International Conference on Bioinformatics and Biomedicine 2012: Proteome Science. The full contents of the supplement are available online at http://www.proteomesci.com/supplements/11/S1.
